# 3D–4D Printed Objects: New Bioactive Material Opportunities

**DOI:** 10.3390/mi8040102

**Published:** 2017-03-27

**Authors:** Céline A. Mandon, Loïc J. Blum, Christophe A. Marquette

**Affiliations:** Univ Lyon, Université Lyon1, CNRS, INSA, CPE-Lyon, ICBMS, UMR 5246, 43, Bd du 11 Novembre 1918, 69622 Villeurbanne CEDEX, France; celine.mandon@univ-lyon1.fr (C.A.M.); loic.blum@univ-lyon1.fr (L.J.B.)

**Keywords:** 3D printing, 4D printing, bioactive materials, enzyme printing

## Abstract

One of the main objectives of 3D printing in health science is to mimic biological functions. To reach this goal, a 4D printing might be added to 3D-printed objects which will be characterized by their abilities to evolve over time and under external stimulus by modifying their shape, properties or composition. Such abilities are the promise of great opportunities for biosensing and biomimetic systems to progress towards more physiological mimicking systems. Herein are presented two 4D printing examples for biosensing and biomimetic applications using 3D-printed enzymes. The first one is based on the printing of the enzymatic couple glucose oxidase/peroxidase for the chemiluminescent detection of glucose, and the second uses printed alkaline phosphatase to generate in situ programmed and localized calcification of the printed object.

## 1. Introduction

A bioactive material is a material with the capacity to actively interact with biological compounds or living organisms [1]. This capacity usually originates from recognition elements integrated within the material, these elements being either biomolecules (enzymes, antibodies, carbohydrate, living cells, etc.), chemical functions (adhesive or repellent surface) or surface microstructures. The active interaction might lead, depending of the field of application of the bioactive material, to a sensing (in the biosensor field) or a mimicking behavior (in the tissue engineering field). When dealing with sensing material, the interaction occurs with a specific analyte and will produce a signal which will be converted into a measurable response [2,3,4]. When dealing with tissue engineering material, the interaction might be specific to the tissue studied and might generate a biomimetic function (elasticity, cellular development, strength, movement, etc.) [5,6,7,8].

Our approach to this vast field of research is to study the possibilities to design “smart” materials based on 4D printing technologies through the direct integration of enzymes during the printing process. This approach enables us to combine all advantages of 3D printing (shape control and complexity) with the numerous possibilities of enzymatic reactions.

The 4D notion was introduced for the first time into the architectural lexicon in 2013, by Skylar Tibbits, co-director and founder of the Self-Assembly Lab hosted by Massachusetts Institute of Technology (MIT)’s International Design Center. This concept introduces the notion of time in 3D printing in order to add new capabilities to multi-material 3D objects. For Skylar Tibbits and his team, but also several other teams involved in the field [9], 4D materials are constructed through 3D printing in such a way that they later react and change shape in response to an external stimulus such as heat or moisture. Their Hilbert Gen 1 Water self-folding tool is one example of such a well-known system [10]. Similar works were performed by Jennifer Lewis’s team at the Wyss Institute for Biologically Inspired Engineering at Harvard University, who proposed several flower-shaped tools, inspired by naturally occurring structures, which respond and change their form in response to environmental stimuli [8]. The team has unveiled 4D-printed hydrogel composite structures that change shape when immersed in water.

**Figure 1:**
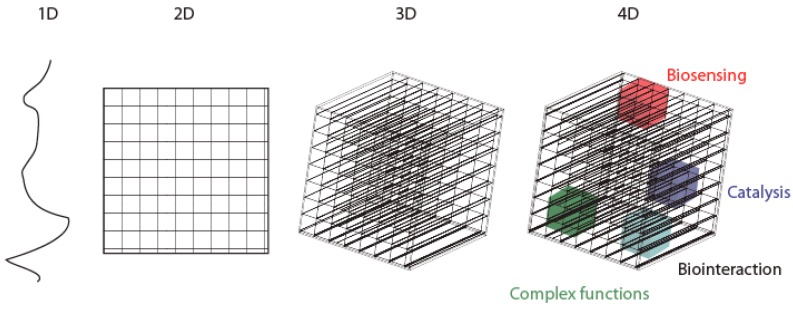
From 1D to 4D concept: 4D printing is adding new capabilities to multi-material 3D-printed objects: either physical, electronic, chemical, biological or biochemical properties.

Our team would like to enlarge this definition so as to not restrict the 4D printing to the ability to program physical and biological materials to change shape, but also to define 4D printing as adding new capabilities to multi-material 3D-printed tools: either physical, electronic, chemical, biological or biochemical properties (Figure 1). This notion is now joined also by the definition of bioprinting that is now widely classified in this 4D printing category, due to its post-print maturation steps that occur over time [11,12,13,14].

In the present report, we will describe two examples of enzymatic reactions that can confer a fourth dimension to a complex 3D-printed shape (fanciful ball). The two chosen reactions are (I) the glucose oxidase/peroxidase sequential reaction and (II) phosphate ester hydrolysis by alkaline phosphatase. These two reactions are chosen examples that will permit us to demonstrate the potentiality of the technique in both the biosensing and tissue engineering fields.

## 2. Materials and Methods

### 2.1. Chemicals

Glucose oxidase from Aspergillus Niger (GOx type X-S), horseradish peroxidase, Alkaline phosphatase (Type I-S, bovine intestinal mucosa), Cresol red, Irgacure 819 (phenyl bis(2,4,6-trimethylbenzoyl)-phosphine oxide), poly(ethylene glycol) diacrylate with mean molecular weight of 700 DA (PEG700DA), luminol (3-aminophthalhydrazide), collagen from calf skin type I, Trizma^®^ base (Tris(hydroxymethyl)aminomethane), calcium chloride, α-d-glucose-1-phosphate (G1P) disodium salt hydrate, silver nitrate and sodium thiosulfate were purchased from Sigma (Lyon, France). Potassium chloride, Veronal (diethylmalonylurea sodium) and sodium phosphate were obtained from Prolabo (Fontenay-sous-Bois, France). Phosphate Buffer Saline (PBS) tablets were purchased from Applichem GmbH (Darmstadt, Germany). All solutions were prepared using milliQ water.

### 2.2. 3D Printing Procedure

**Figure 2:**
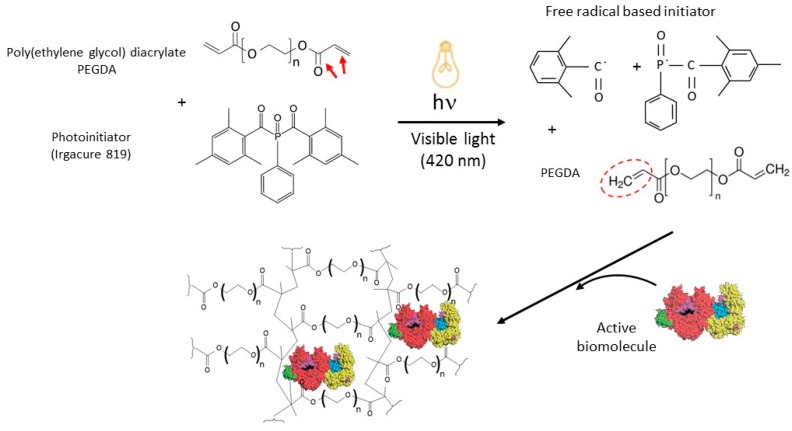
Hydrogel 3D printing process. Poly(ethylene glycol) diacrylate photopolymerization reaction and inclusion of biomolecules.

Plain printing ink was prepared by mixing 2.5 mL of aqueous solution of cresol red at a concentration of 250 µg/mL with 1.5 mL of PEG700DA and 150 µL of Irgacure 819 at a concentration of 10 mg/mL in ethanol. Once this solution prepared, the different biomolecules were added at final a concentration of: 1.25 µg/mL of peroxidase and 1.25 µg/mL of glucose oxidase; 2.5 µg/mL of peroxidase; 2.5 µg/mL of glucose oxidase; 0.05 mg/mL of collagen and 2.5 µg/mL of alkaline phosphatase (Figure 2).

**Figure 3:**
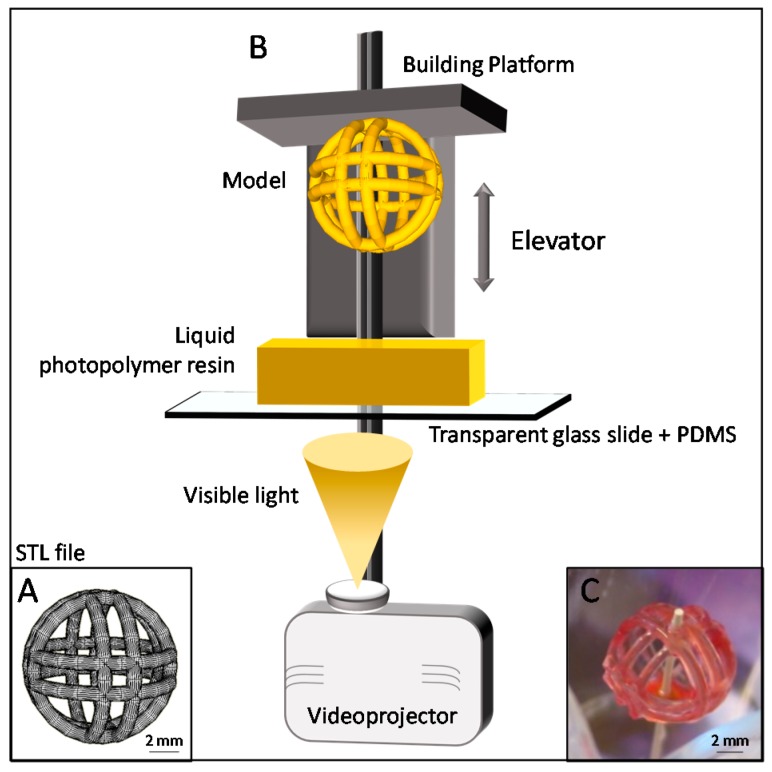
Fanciful ball printing. The digital light processing (DLP) printing set-up and concept. Images of each layer are projected in a liquid resin batch to generate polymerization. Additive manufacturing process—Fanciful ball. (**A**) Standard Tessellation Language (STL) file; (**B**) DLP process; (**C**) 3D-printed object.

The printing procedure was performed using a B9Creator open source system (B9Creation, Rapid City, SD, USA): a digital light processing (DLP)-based process using with visible light irradiation. For these experiments, a conventional light source projects an entire slice/layer of the model with a slicing thickness of 101.6 µm (Figure 3). All 3D computer-aided design (CAD) files were generated using SketchUp 8 (Trimble Navigation Limited, Sunnyvale, CA, USA). The hardware parameters adapted to the present ink formulation were: attach first two layers exposure time 30 s, initial image exposure time 15 s and perimeter exposure time 4 s.

For multi-components printing, different tanks of resin were successively used with rising of the object and washing using PBS buffer.

### 2.3. Enzyme Assays

All enzyme assays were performed at room temperature.

#### 2.3.1. Chemiluminescent Assay

Chemiluminescent assays were performed in the presence of luminol 220 µM and glucose 100 mM in a pH 8.5 buffered solution composed of Veronal at a concentration of 30 mM and KCl at a concentration of 30 mM (VBS). Fanciful balls were immersed in VBS containing both substrates and the chemiluminescent signal imaged using a charged coupled device (CCD) cooled camera (Las-1000 Plus, Intelligent Dark Box II, FUJIFILM, Tokyo, Japan). Integration times are indicated in minutes on result pictures.

#### 2.3.2. Calcification Process

Calcifications were performed in the presence of calcium chloride at a concentration of 15 mM and G1P at a concentration of 20 mM in a pH 9 buffered solution composed of Trizma^®^ at a concentration of 20 mM. Fanciful ball composed of a half part of plain hydrogel and the other half part containing alkaline phosphatase was immersed into 5 mL of this buffered solution during seven hours.

Visualization of calcium precipitate is performed using the Von Kossa staining [15]. For that purpose, fanciful balls are treated with a 1% silver nitrate solution and expose to a ultraviolet (UV) light during 5 min. Then the fanciful ball is rinsed with milliQ water before a washing step into a solution containing 5% of sodium thiosulfate during 20 min. The fanciful balls were then rinsed into water before observation and imaging using inverted microscopy (Olympus, Rungis, France).

## 3. Results

This study on the enzymatic activities of 3D-printed objects is focused on one single form, the fanciful ball, with a complex and precise structure. The DLP printing process and customization of our B9 creator printer allow the stratification of several ink formulations. Indeed, we designed ink containers that can receive several ink formulations to reduce their size and adapt their shape to the application (CAD files of the B9 modification can be found in Appendix A). Hydrogel changes are then possible during the printing process and inks can be tuned by mixing polymers or by adding different biomolecules into the Polyethylene glycol diacrylate (PEG-DA) hydrogels. In that way, entrapped enzymes can be printed within a unique section of the printed object, or separated in several sections of the object, in order to catalyze sequential reactions. Biomolecules were physically trapped within the PEG acrylate network. Meshes of sufficient size are produced using PEG700DA to trap the enzyme while still giving the possibility to the substrates to diffuse within the hydrogel.

We chose to use very well-characterized enzymes as model systems: glucose oxidase (GOD) combined with horseradish peroxidase (HRP) or alkaline phosphatase. These enzymes have potential practical applications in clinical and pharmaceutical analysis (glucose detection, labeling for immunoassays, etc.) and were herein co-immobilized or separately immobilized in the fanciful balls to demonstrate the capacity of the system to generate a 4D-printed object with smart functions.

Numerous other objects can be printed with the developed 4D printing technique [3] but the printed object shape selected for the present study was the fanciful ball depicted in Figure 3A. This object was chosen for its very challenging shape, i.e., airy structure (structure is 1 mm in diameter) with thin edifices but still a large size of the complete object. This object also presents a Janus symmetrical structure that we believe might be of interest in sequential enzymatic reactions (not demonstrated yet).

**Figure 4:**
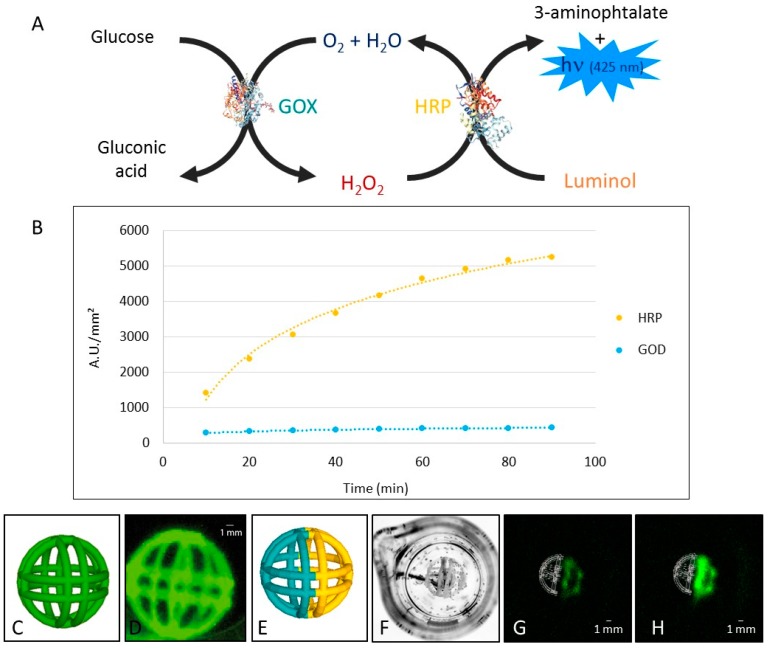
Bi-enzyme chemiluminescent system. (**A**) Glucose oxidase and horseradish peroxidase are working sequentially to produce light in the presence of glucose and luminal; (**B**) chemiluminescent signal over time. Yellow curve corresponds to the signal measures on the horseradish peroxidase (HRP) half-ball and the blue one to the signal measures on the glucose oxidase (GOD) half ball; (**C**) a homogeneous fanciful ball composed of glucose oxidase and peroxidase is used; (**D**) chemiluminescent image of the homogeneous fanciful ball in the presence of glucose 100 mM and luminol 220 µM (180 s integration); (**E**) a heterogeneous fanciful ball composed of glucose oxidase (left) and peroxidase (right) is used; (**F**) visible light image of the heterogeneous fanciful ball; (**G**) chemiluminescent image of the homogeneous fanciful ball in the presence of glucose 100 mM and luminol 220 µM (600 s integration, 60 and 90 min after substrates addition left (**G**) and right (**H**), respectively).

For the first exemplary study we explored the feasibility of co-immobilizing two enzymes, glucose oxidase and horseradish peroxidase, within a unique printed object. The chemiluminescent signal generated in the presence of luminol and glucose was thus used as an indicator of the enzymes’ activity and sequential work (Figure 4A), i.e., efficient diffusion of one enzyme reaction product to the second enzyme’s catalytic center.

As shown in Figure 4C, the addition of both enzyme substrates, glucose and luminol, triggers the sequential reaction and induces light emission from the 4D-printed object (Figure 4B). Moreover, the details of the fanciful ball, a 1-cm-diameter ball composed of 1-mm-diameter crossing structures, appeared clearly in the obtained chemiluminescent images, demonstrating the homogeneous distribution of both enzymes within the structure.

The second step of the exemplary study was performed by separating those two enzymes in two distinct parts of the object (Figure 4D). In the left-hand side of the fanciful ball, glucose oxidase was entrapped in the PEG hydrogel during the printing process. Then, horseradish peroxidase was entrapped into the polymer on the right-hand side of the ball. This approach then generates a spherical bi-enzyme object with a defined shape and enzyme distribution.

One more time, the addition of glucose and luminol into the reaction medium induced a chemiluminescent light emission, proof of the successful sequential enzymatic reactions. Moreover, in this second set-up, since enzymes were physically separated in two distinct parts of the printed object, the immediate light emission was localized only within the peroxidase-modified part (Figure 4F). Increasing the reaction time (Figure 4G) led to an accumulation of hydrogen peroxide within the ball, evidenced by a strong increase of the chemiluminescent signal.

**Figure 5:**
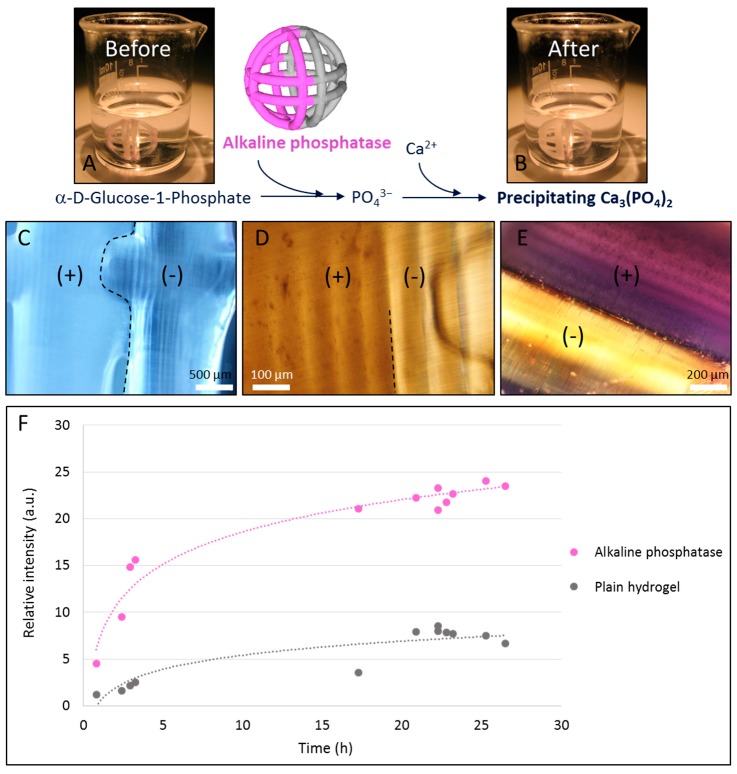
In situ calcification system. (**A**) A heterogeneous fanciful ball composed of alkaline phosphatase (left) and plain hydrogel (right) is used; (**B**) heterogeneous fanciful ball after reaction with α-d-glucose-1-phosphate; (**C**,**D**) microscopic view of the limit between the enzyme and plain hydrogel parts; (**E**) microscopic view of the limit between the enzyme and plain hydrogel parts after Von Kossa staining; (**F**) calcification process over time.

In a third exemplary study, the fanciful ball was printed in the presence of alkaline phosphatase, subsequently used to generate in situ calcification of the hydrogel, and then demonstrated the interest of the technique for tissue engineering (in bone reconstruction for example). The enzyme-based in situ calcification was based on the catalysis of the hydrolysis of the α-d-glucose-1-phosphate by the alkaline phosphatase which generates phosphate. This phosphate, in the presence of free calcium, rapidly forms calcium phosphate precipitate on the printed structures (Figure 5A). Similarly to the latter study, the fanciful ball was composed of two parts, the first one containing the alkaline phosphatase and the second one composed of plain hydrogel (Figure 5A). Type I collagen was also added into the structure of the hydrogel to enable the structuration of the calcium phosphate [16,17].

Macroscopic observation of the fanciful ball before (Figure 5A) and after (Figure 5B) in situ precipitation revealed a strong color change, from translucent to foggy white, of the alkaline phosphatase part of the object. This color difference was also observed at the microscopic level (Figure 5C–E) with a high control of the precipitation down to the single printing layer resolution (100 µm). A higher microscopic magnification shows that the structure containing the enzyme exhibits aggregates which may correspond to calcium precipitates (Figure 5D).

In order to clearly demonstrate the formation of calcium phosphate within the printed object, a Von Kossa coloration was performed [18] which enabled the purple staining of the precipitate. As we can see in Figure 5E, Von Kossa staining was clearly observed again with a single printed layer (100 µm) resolution of the calcification.

## 4. Conclusions

The present study is only the third report [3,19] about direct enzyme integration into 3D printing, i.e., 4D printing, and the second about DLP 3D-printed objects. A demonstration was made by our group in a previous publication [3] showing that the concept is powerful, and the present report has focalized on a single object used in two different applications: biosensing and tissue engineering.

Two major results were obtained here using the fanciful ball’s highly challenging 3D-printed shape. First, sequential enzymes were shown to be able to be retained in such an airy 3D architecture and worked sequentially to generate light in the presence of glucose. The foreseen impact of such a technique is without a doubt in the field of smart materials for biosensors where these new 3D sensing layers shall demonstrate new properties in the near future [20].

The second result was the programmed calcification of a 4D-printed object with high resolution and accuracy thanks to the printing of alkaline phosphatase–modified 3D ink. This strategy opens the path to a complete new field of research in tissue engineering and particularly bone reconstruction, in which cells and programmed calcification might be printed together to enable the in vitro reconstruction of cellularized bone defects. Going further in the analysis of the impact of such technology, more tissue engineering applications might be impacted, such as vascularization [21], brain tissue production [22], and more generally multi-tissue 4D printing through the use of printed enzymes.

## Appendix A

CAD files necessary to the modification of the B9 Creator DLP printer are available in https://gist.github.com/FabricAdvancedBiology.
